# Flooding in Townsville, North Queensland, Australia, in February 2019 and Its Effects on Mosquito-Borne Diseases

**DOI:** 10.3390/ijerph16081393

**Published:** 2019-04-17

**Authors:** Adeshina I. Adekunle, Oyelola A. Adegboye, Kazi Mizanur Rahman

**Affiliations:** 1 Australian Institute of Tropical Health and Medicine, James Cook University, Townsville, QLD 4811, Australia; adeshina.adekunle@jcu.edu.au (A.I.A.); kazi.rahman@jcu.edu.au (K.M.R.); 2 College of Medicine and Dentistry, Division of Tropical Health and Medicine, James Cook University, Townsville, QLD 4811, Australia

**Keywords:** flooding, mosquito-borne diseases, North Queensland, dengue, Ross River virus, wet–dry tropics

## Abstract

In February 2019, a major flooding event occurred in Townsville, North Queensland, Australia. Here we present a prediction of the occurrence of mosquito-borne diseases (MBDs) after the flooding. We used a mathematical modelling approach based on mosquito population abundance, survival, and size as well as current infectiousness to predict the changes in the occurrences of MBDs due to flooding in the study area. Based on 2019 year-to-date number of notifiable MBDs, we predicted an increase in number of cases, with a peak at 104 by one-half month after the flood receded. The findings in this study indicate that Townsville may see an upsurge in the cases of MBDs in the coming days. However, the burden of diseases will go down again if the mosquito control program being implemented by the City Council continues. As our predictions focus on the near future, longer term effects of flooding on the occurrence of mosquito-borne diseases need to be studied further.

## 1. Introduction

**Figure 1:**
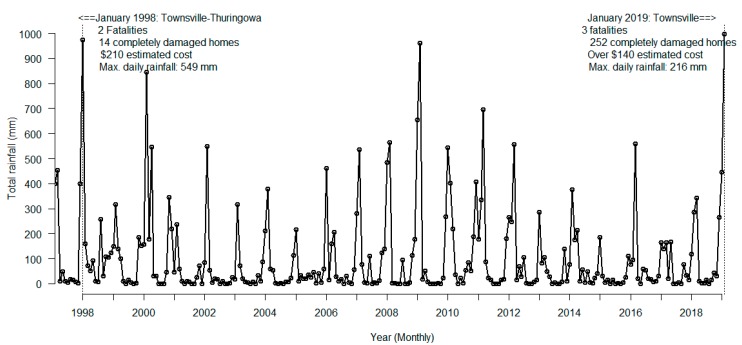
Total monthly rainfall in Townsville area. The dotted line indicates flooding events.

The major inundation that happened in the first week of February 2019 in Townsville resulting from heavy monsoonal rain and overflowing of the Ross River dam was unprecedented [1]. A large portion of the built areas in this North Queensland capital city of Australia was inundated, especially when the flooding reached its peak on the evening of 5 February 2019 after all the spillway gates of the Ross River dam were opened, as the dam reached more than 200% of its capacity (see Figure 1 for total monthly rainfall since 1998). As the rainfall has now stopped, the water level has subsided in the river, and the inundation has receded, the residents of the affected suburbs are now gradually returning to their properties. This event may expose them to different health problems, including vector-borne diseases, especially those transmitted by mosquitoes.

We predicted the changes in the occurrence of mosquito-borne diseases (MBDs) in Townsville in the coming days as a result of the flooding. We reviewed the existing literature on flooding and increases in MBDs in the context of the area. We also did mathematical modelling to predict the changes in the occurrence of MBDs in Townsville in the coming days.

In Townsville, Ross River virus disease (RRV), Barmah Forest virus (BFV) disease, and dengue are the prevalent mosquito-borne diseases [2]. The *Aedes* and *Culex* mosquito populations are predominant in this area. While *Aedes* mosquitoes breed in fresh stagnant water in and around the dwellings or in marshlands, *Culex* population grow in poorly kept sewerage, and farms or irrigated fields with water left standing for several days [3,4]. The role of flooding on the increased occurrence of dengue, BFV disease, and RRV disease in the context of Townsville is arguable. Previous studies have found a significant association between incidence of flooding and upsurge of infectious diseases [5,6,7,8,9]. While flash flooding initially disturbs the mosquito habitat, this may or may not subsequently increase the occurrence of mosquito populations and MBDs in the flood-affected communities [8,10,11]. This will depend on the specific MBD we are concerned about and its local endemicity. If it is dengue, which is primarily transmitted locally from imported cases, this is less likely. Other than the returnees from overseas, especially those from the Southern Hemisphere, where dengue season is currently occurring, the current flooding scenario in Townsville will attract very few tourists who could bring the disease with them. However, in considering flooding events in tropical settings in other parts of the world, a positive association has been observed between river level and dengue incidence [10].

RRV disease is more common in the Townsville area and is in fact named after the river that primarily caused the recent major flooding. RRV disease has been found to be associated with flooding in previous studies [9] and its spread in this region will depend on the availability of the animal reservoir from where the vector mosquitoes are infected in addition to infected humans. Major flooding can destabilize and displace animal populations, including kangaroos and wallabies, which could carry the virus and lead to mosquitoes-borne transmission to humans [11]. However, the inundation could also bring the animals closer to humans, as the animals may take shelter closer to and inside of the unflooded human habitats.

## 2. Materials and Methods

### 2.1. Study Area and Data Sources

**Figure 2:**
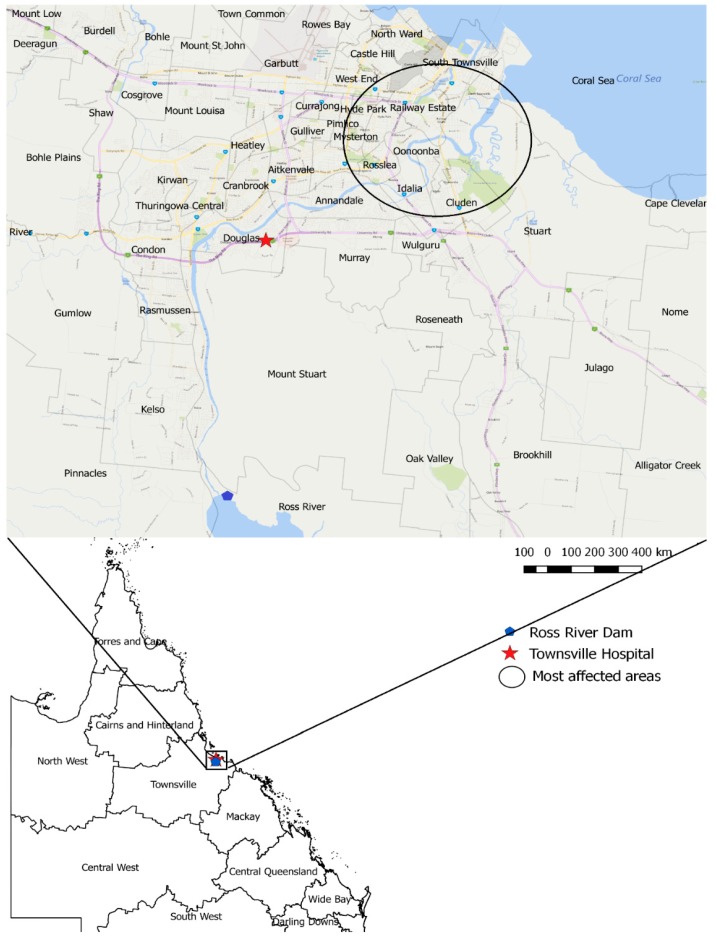
Map of Townsville township area indicating the Townsville Hospital, the Ross River Dam, the Ross River, and its pathway through the suburbs of the city to the sea.

Townsville is located in the wet–dry tropics, in the north-east coastal region of Australia (Figure 2), which is characterized by high temperatures throughout the year [12] with the majority of the rainfall generated by monsoonal lows and thunderstorms during the summer season [13]. According to the 2016 census, there were about 226,031 people residing in Townsville [14]. The number of notifiable cases of mosquito-borne diseases reported to Queensland Health [15] in 2018 was 310, with the majority reported as RRV infections.

### 2.2. Mathematical Models

With the floodwaters having just receded, its impacts are still being felt, and the likelihood that this event may lead to an increase in the number of arboviral infections is not clear [11,15]. We developed a mathematical model of transmission of MBDs to study the possibility of an increase in the number of these diseases in the coming weeks. The minimal models for the dynamics of MBDs are an SI ($S_{H}$—number of susceptible people, $I_{H}$—number of infectious people) for the human population and an SI ($S_{M}$—number of susceptible mosquitoes, $I_{M}$—number of infectious mosquitoes) for the mosquito population (pp. 70–71, [16]). Hence, we have:

(1) $$\frac{{\mathrm{dS}}_{H}}{\mathrm{dt}}=\mu_{H}N_{H}-\frac{{\alpha \beta I}_{M}S_{H}}{N_{H}}-\mu_{H}S_{H}+{\gamma I}_{H}\;\frac{{\mathrm{dI}}_{H}}{\mathrm{dt}}=\frac{{\alpha \beta I}_{M}S_{H}}{N_{H}}-{\gamma I}_{H}-\mu_{H}I_{H}\;\frac{{\mathrm{dS}}_{M}}{\mathrm{dt}}=N_{M}\left(\pi_{M}-\frac{\mu_{M}S_{M}}{K_{\mathrm{BF}}}\right)-\frac{{\alpha \beta I}_{H}S_{M}}{N_{H}}$$

(2) $$\frac{{\mathrm{dI}}_{M}}{\mathrm{dt}}=\frac{{\alpha \beta I}_{H}S_{M}}{N_{H}}-\frac{\mu_{M}N_{M}I_{M}}{K_{\mathrm{BF}}}$$

From Equation (2), the dynamics of the mosquito population is:

(3) $$\frac{dN_{M}}{dt}=N_{M}\left(\pi_{M}-\frac{\mu_{M}N_{M}}{K_{BF}}\right)$$

and the human population is constant (say$\;N_{H}$). See Table 1 for descriptions of the parameters used for the simulation of MBDs. The parameter choices are based on the fact that the arboviral infections have similar infection dynamics; especially, RRV and BFV are indistinguishable except for serological diagnosis [17]. For dengue, disease importation from overseas is not common in Townsville and when it happens the disease prevention strategies can effectively contain the case(s) and control the transmission locally [18]. For RRV and BFV, they both depend on the infected mosquito population and their ability to transmit the viruses.

From Table 1, it can be shown that $\frac{I_{H}}{N_{H}}\ll 1$ and $N_{M}\approx S_{M}$, where $N_{M}$ is the total mosquito population. $N_{M}\approx S_{M}$ is a reasonable assumption to make, even when the infected mosquitoes cannot transmit viruses. With this information, the number of infected mosquitoes is approximately:

(4) $$I_{M}=\frac{\alpha \beta I_{H}K_{BF}}{\mu_{M}N_{H}}$$

This shows that the infected mosquitoes are dependent on the carrying capacity ($K_{BF}$), which increases as a result of flooding. Also, with $S_{H}\approx N_{H}$, the number of infected people is approximately:

(5) $$I_{H}\approx \min\left(I_{H}^{0}e^{\left(\frac{\alpha^{2}\beta^{2}K_{BF}}{\mu_{M}N_{H}}-\gamma -\mu_{H}\right)t},N_{H}\right)$$

**Figure 3:**
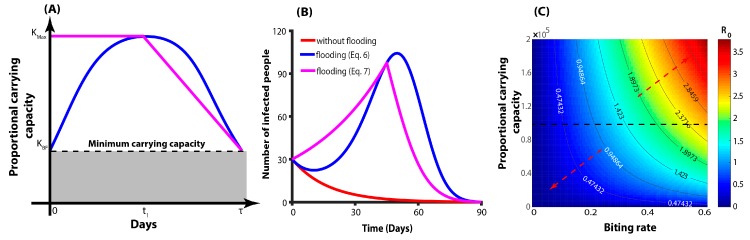
(**A**) Schematic representations of the form of the carrying capacity as a result of the flooding. The blue line denotes the carrying capacity form in Equation (6) and the pink line for Equation (7). (**B**) Predicted number of infected people under different scenarios. (**C**) Relationship between biting rate, carrying capacity and the basic reproduction number. The red arrows show the direction of increase and decrease of the$\;R_{0}$. If people are more protected from bites then no endemic situation will be observed.

Furthermore, given that in the wake of the current flooding event the Townsville Council is intensifying its vector control programs, we will expect that the additional number of breeding sites as a result of flooding returns to the minimal level $K_{BF}$ say in time τ days. Hence, we can represent the form of proportional carrying capacity due to flooding by these equations (Figure 3A):

(6) $$K_{F}=K_{BF}+(K_{max}-K_{\mathrm{BF}})\sin\left(\frac{\pi t}{\tau }\right)$$

(7) $$K_{F}=\{\begin{array}{c} K_{max},\;\;\;\;\;\;\;\;\;\;\;\;\;\;\;\;\;\;\;\;t\leq t_{1}\\ K_{max}+\frac{(K_{BF}-K_{max})\left(t-t_{1}\right)}{\tau -t_{1}},\;t_{1}<t\leq \tau \end{array}$$

where $K_{max}$ is the proportional maximum carrying capacity as a result of the flooding. From Equations (1) and (2), the basic reproduction number is:

(8) $$R_{0}=\alpha \beta \sqrt{\frac{K_{BF}}{N_{H}\mu_{M}\left(\gamma +\mu_{H}\right)}}$$

Also, this is dependent on the carrying capacity and an increase in the carrying capacity may increase the number of infected people. However, it is clear from the current disease burden in Townsville that $R_{0}<1\;$ (assume 0.83), which could be attributed to the recent roll out of the *Wolbachia* method in the region and its effect on MBD transmission [19,20]. We also conducted a sensitivity analysis to examine the impacts of the variability in the initial number of people infected, biting rates and transmission probability (due to *Wolbachia* roll-out in Townsville [19]) on the peak of the epidemic curve.

## 3. Results

We begin our analysis by setting the time period ($t_{1}$) to 90 days after the flooding and two forms of the carrying capacity ($K_{F})$. Our modelling results showed the contribution of the mosquito carrying capacity on the basic reproduction number ($R_{0})$. Figure 3A presents the schematic representations of the two forms of the carrying capacity based on three parameters: the mosquitoes’ carrying capacity before the flooding $K_{BF}$, the maximum carrying capacity as a result of flooding $K_{max}$, and the study period τ. We believe that this model adequately captures the exposure probability of infection before and after the flooding, which is very difficult to measure in an unbiased way.

Figure 3B shows the resulting predictions for $I_{H}$ based on the two forms of $K_{F}$. Using the 2019 year-to-date (YTD) number of notifiable MBDs (30 people as of 7 February 2019) [15] resulted in an increased number of infections (peaked at 104 (Equation (6)) and 96 (Equation (7))) that later reduces to zero (Figure 3B). Without flooding, the number of infected people goes down to zero because of minimal model and $R_{0}<1$, and we observed a sharp increase due to an increase in the carrying capacity as a result of flooding. We further examined how biting rate as a determinant of exposure and the mosquito carrying capacity would affect the basic reproduction number. As expected (Figure 3C), increasing the biting rate and mosquito carrying capacity will lead to an increase in the basic reproduction number, thus resulting in more people being infected and the MBDs becoming endemic. Importantly and irrespective of the level of the mosquito carrying capacity, there is a minimal biting rate at which the basic reproduction number is still less than one. However, with a lower mosquito carrying capacity and higher exposure to infective bites the basic reproduction number can still be greater than one.

**Figure 4:**
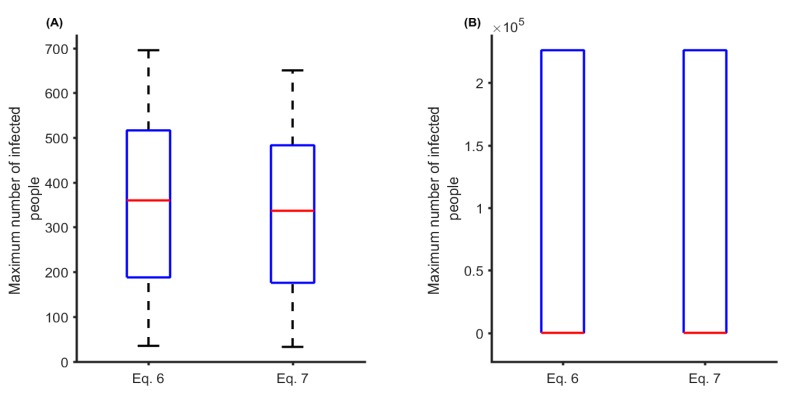
(**A**) The effects of the initial number of infected people on the peak of the epidemic curve. In (**A**), we assume $I_{H}^{0}$ is uniformly distributed with integer random numbers between 10 and 200 inclusively. (**B**) The variability in the peak of infection due to varying biting rate (α∈U(0.01, 3.75)) and transmission probability (β∈U(0.01, 0.75)). In this case, the initial number of infected people is 30 based on the year-to-date (YTD) number of notifiable mosquito-borne diseases (MBDs) in Townsville as of 7 February 2019 [15].

In Figure 4A, we demonstrated how the initial number of infected people could change the course of the infection dynamics. For this simulation, the median peaks of people infected with MBDs are 358 and 335 for Equations (6) and (7), respectively. When we vary the biting rate and transmission probability, this implies that $R_{0}$ can be greater than one following Figure 3C and hence everyone is infected and the MBDs persist (Figure 4B).

## 4. Discussion

In this study, we evaluated the impacts of flooding on mosquito-borne diseases in Townsville, North Queensland, Australia, and determined whether this situation will lead to serious health consequences. As the major mosquito-borne diseases are effectively controlled by the health authorities and through sensitization of residents, the spread of the diseases are kept under control before flooding events and this is expected to continue after flooding events as well. We have used parameters that ensure the basic reproduction number is less than one, as expected in Townsville, and if this is maintained, only a gradual increase in the number of infected persons will be noticed and over the long term the burden will reset to its level before the flooding event Otherwise, similar to the 2008/2009 Cairns, Queensland, Australia dengue epidemic, where the basic reproduction number was estimated to be between 2 to 12 for the first two months of the epidemic [21], Townsville may face an elevated burden of disease that will persist for a longer period of time. However, the scenario will not be the same as the one in Cairns a decade ago, as the *Wolbachia* method*,* a vector control method to control for arboviral infections, has been rolled out in the region including Cairns, Townsville, and their neighboring areas [19]. Furthermore, a portion of the population in this area may be immuned to these endemic vector-borne diseases. All these factors will eventually affect the assumptions behind calculating the basic reproduction number, which usually applies to a completely susceptible population. Hence, the assumption of an effective reproduction number less than one in the context of Townsville region is valid because the calculation of basic reproduction number usually applies to a completely susceptible population.

This study is subject to a number of limitations. First, we restricted our projection to 90 days after the flooding event; further studies may need to be carried out to determine whether or not this is a sufficient number of lag days. Also, there can be other factors, like king tides, which may affect the low-lying areas in the coming days, including marshlands, resulting in further inundation and increased breeding of mosquito populations. In addition, we did not consider any delayed exposure effect in this study. Lastly, we have made a few assumptions regarding the parameters used based on literature, especially in regard to $R_{0}$, which may be either much lower or closer to one than expected. This implies that we may have either less or more people infected in the period following the flooding event before the flood effects on MBDs normalize. Also, when $R_{0}<1$ in most cases it implies that eventually the number of people infected will decrease to zero. As the dynamics of these MBDs are subjected to random events, and follow a stochastic pattern, $R_{0}<1$ does not guarantee a disease-free state [27]. Even in deterministic fashion, the concept of backward bifurcation implies that there are values for $R_{0}<1$ at which the disease does not die out [28]. Hence, analysis of a detailed model may not give zero infected people after 90 days as predicted by our model.

## 5. Conclusions

The results from this study have consistently shown that a flooding event is likely to increase the number of mosquito-borne infections and increase the carrying capacity of the vector population. Our forecast has demonstrated the importance of putting in place control measures and elevating public health awareness to reduce the potential spread of the diseases. The focus should be on the removal of mosquito breeding sites, including fresh stagnant or stored water in and around household dwellings. This applies to the accommodations where the flood-affected residents relocated to on a temporary basis. This also applies to the condition of the inundated houses in the period surrounding the return of the residents. An effective mosquito surveillance program is needed to be put in place in order to identify newly developed mosquito breeding sites and intervene when the mosquitoes are still at the larval stage. All these actions should help to eventually decrease the carrying capacity of the mosquitoes and thus reduce the transmission of the mosquito-borne diseases. In addition, personal protection against exposure to mosquito bites via the use of window screens, mosquito repellents, and avoiding mosquito bites at peak times like at dawn or dusk will also be beneficial and help to reduce the mosquito-borne disease transmission.

In the near future when adequate data become available, further studies incorporating lag days may achieve a better forecast and explain the delayed exposure effect of flooding.

## Figures and Tables

**Table 1:** Parameter descriptions and values for model Equations (6) and (7).

Parameter	Description	Value (Range)	Unit	References
β	Transmission probability	0.25–0.75	Dimensionless	[21,22,23]
α	Biting rate	0.172–0.375	Person per mosquito per day	[21,24]
$\pi_{M}$	Mosquito birth rate	0.09	Per day	[25]
$K_{BF}$	Proportional carrying capacity	100,000	Mosquitoes	Estimated
$K_{max}$	Maximum proportional carrying capacity due to flooding	200,000	Mosquitoes	Assumed
$N_{H}$	Total population	226,031	People	[16]
τ	Time until carrying capacity normalizes	90	Day	Assumed
γ	Recovery rate	0.143–0.22	Per day	[21,24]
$\mu_{H}$	Mortality rate	0.000034	Per day	[26]
$\mu_{M}$	Mosquito death rate	0.026–0.036	Per day	[21]
